# The Role of Autophagy in Liver Cancer: Crosstalk in Signaling Pathways and Potential Therapeutic Targets

**DOI:** 10.3390/ph13120432

**Published:** 2020-11-28

**Authors:** Jianzhou Cui, Han-Ming Shen, Lina Hsiu Kim Lim

**Affiliations:** 1 Department of Physiology, Yong Loo Lin School of Medicine, National University of Singapore, Singapore 117593, Singapore; phscui@nus.edu.sg; 2 NUS Immunology Program, Life Sciences Institute, National University of Singapore, Singapore 117456, Singapore; 3 Faculty of Health Sciences, University of Macau, Macau, China; 4 NUS Graduate School for Integrative Sciences and Engineering, National University of Singapore, Singapore 119077, Singapore

**Keywords:** autophagy, HCC, ULK1, mTOR, AMPK, MAPK, p53

## Abstract

Autophagy is an evolutionarily conserved lysosomal-dependent pathway for degrading cytoplasmic proteins, macromolecules, and organelles. Autophagy-related genes (Atgs) are the core molecular machinery in the control of autophagy, and several major functional groups of Atgs coordinate the entire autophagic process. Autophagy plays a dual role in liver cancer development via several critical signaling pathways, including the PI3K-AKT-mTOR, AMPK-mTOR, EGF, MAPK, Wnt/β-catenin, p53, and NF-κB pathways. Here, we review the signaling pathways involved in the cross-talk between autophagy and hepatocellular carcinoma (HCC) and analyze the status of the development of novel HCC therapy by targeting the core molecular machinery of autophagy as well as the key signaling pathways. The induction or the inhibition of autophagy by the modulation of signaling pathways can confer therapeutic benefits to patients. Understanding the molecular mechanisms underlying the cross-link of autophagy and HCC may extend to translational studies that may ultimately lead to novel therapy and regimen formation in HCC treatment.

## 1. Introduction

Autophagy is a self-degradative process that represents an important physiological catabolic mechanism of the eukaryotic cell. It is hallmarked by a lysosome-dependent process which allows the degradation and recycling of cellular components, such as cellular organelles and macromolecules, in a given order. During autophagy, the autophagosome, a double-membrane vesicle structure, engulfs portions of the cytoplasm and subsequently fuses with lysosomes to form autolysosomes for further degradation [1,2,3,4,5,6,7]. In eukaryotic cells, there are three major types of autophagy: macroautophagy, microautophagy, and chaperone-mediated autophagy (CMA). Macroautophagy (hereafter referred to as “autophagy”) is the main pathway, characterized by a autophagosome-autolysosome process and highly conserved autophagy-related genes (Atgs) in the core autophagic machinery. Since the discovery of the first Atg in yeast in 1996 [8], more than 40 Atgs and Atg homolog genes have been identified across different species [9,10]. The liver is essential for the maintenance of metabolic homeostasis, and thereby autophagy plays a vital role in the organismal energetic balance. Energy homeostasis mainly relies on cytosolic and organelle protein degradation and amino acid recycling by autophagy in the liver. Besides protein breakdown, hepatic glycogen stores become an essential energy source when no longer available through dietary intake. The hydrolases present in lysosomes are capable of degrading proteins, carbohydrates, lipids, and nucleic acids. Moreover, the autophagic adjustment of the mitochondrial metabolic capacity in the liver further supports the notion that hepatic autophagy contributes to liver metabolism [11].

It has been widely established that autophagy plays a dual role in cancer, and it has been extensively reviewed [12,13,14,15,16,17,18]. On the one hand, autophagy can function as a tumor-suppression mechanism in the early stage of cancer development by inhibiting inflammation and promoting genomic stability [19]. In vitro and in vivo studies have demonstrated that autophagy could suppress tumorigenesis by removing oncogenic protein, maintaining genomic stability, promoting cell death, inducing stress and immune response, and suppressing reactive oxygen species (ROS) [17,20,21,22,23,24,25,26]. Notably, many autophagy-related genes play an essential role in the suppression function of autophagy tumor development. Autophagy was assigned a tumor suppressive role by the discovery of Beclin1 deletions in multiple tumor types, which demonstrated the tumor-suppressive function of autophagy for the first time [27,28,29]. The mutation of several other autophagy genes, including ATG2B, ATG3, ATG4, ATG5, ATG9B, and ATG12, was also found, together suggesting that autophagy contributes to the suppression role of tumorigenesis [30,31,32,33,34]. While Atg5 and/or Atg7 deletion have been shown to enhance tumor incidence during the early stages of tumorigenesis via RAS signaling, there was no effect on tumor development in the late stage. However, studies have revealed that the loss of autophagy upon Atg5 or Atg7 deletion could accelerate tumor formation in mice containing oncogenic Kras and lacking p53 [35,36,37,38,39]. Hence, autophagy plays an essential role in tumor suppression, and autophagy deficiency or inhibition may contribute to tumorigenesis, especially at the initial stage.

On the other hand, autophagy can promote tumorigenesis by supplying nutrients and energy, reducing hypoxia and oxidative stress, and promoting angiogenesis. Autophagy may also promote tumor metastasis and invasion, regulating unfolded protein response (UPR) as well as providing drug resistance to cancer cells [18,25,39,40,41,42,43,44]. Interestingly, once the tumor has been established, autophagy can help cancer cells cope with environmental stresses. Thus, autophagy is critical for cancer cells’ survival and growth. Many reports have revealed that the genetic ablation of essential autophagy genes in established tumors could inhibit tumor growth, survival, and malignancy [23,38,45,46,47,48,49,50]. Moreover, the deletion of Atg5 or Atg7, atg13 or Ulk1, Fip200, and Atg7 decrease tumor progression in various oncogene-driven cancer types [49,51,52,53,54]. These findings collectively suggest that cancer cells benefit from autophagy activation, while suppressing autophagy inhibits tumor progression and increases tumor cell death [12,17,55].

Hepatocellular carcinoma (HCC) is the most common type of primary liver cancer and the fifth leading cause of death worldwide. The role of autophagy underlying HCC initiation and development has been well studied because autophagy can be induced in hepatocytes and the liver is an important metabolic organ [11,56,57]. Several lines of evidence have linked autophagy to a tumor suppression role in HCC [21,58,59]. For instance, the systemic mosaic deletion of Atg5 and liver-specific Atg7^−/−^ has been shown to cause benign liver adenoma development [31], while Beclin-1 haploinsufficiency induces spontaneous HCC formation [29]. On the other hand, autophagy may protect tumor cells by providing extra arginine in the microenvironment of a liver-specific *Atg7* or *Atg5* deletion mice model, thereby indicating that autophagy partially compensates for nutrient loss in such condition and thus promotes cell growth [47,60]. Changes in autophagic signaling may also impair the metabolic balance of energy and nutrients and seriously impact liver physiology and disease [56,61,62]. The elucidation of the molecular mechanisms underlying autophagy-dependent pathogenesis could lead to the discovery of novel therapeutic approaches for HCC. Evidence has revealed that the suppression or induction of autophagy by the modulation of the mTOR, AMPK, and MAPK pathways plays a critical role in liver tumor development [63,64,65]. Hence, the modulation of autophagy via the key signaling pathways is a promising approach for enhancing the efficacy of existing liver cancer therapies [14,16,58,66,67].

In our previous review, we described the role of autophagy in the physiology of the liver and etiological factors of HCC. The dual role of autophagy in hepatocarcinogenesis and the modulation of autophagy as a novel strategy for liver cancer therapy was also discussed [68]. To further summarize the current progress in the study of autophagy in liver cancer, we will illustrate the regulatory machinery of autophagy and focus on the signaling pathways that link the function of autophagy in HCC development. Following this, we then discuss the potential therapeutic approaches for HCC treatment by targeting those pathways involved. We hope to provide a comprehensive view and reference for a deep understanding of the underlying mechanisms in the role of autophagy in liver cancer and a unique visual angle for HCC treatments involving autophagy modulation as an HCC therapeutic regimen strategy.

## 2. The Role of Autophagy in HCC via Autophagy Core Machinery Genes

### 2.1. The Core Molecular Machinery of Autophagy

**Figure 1:**
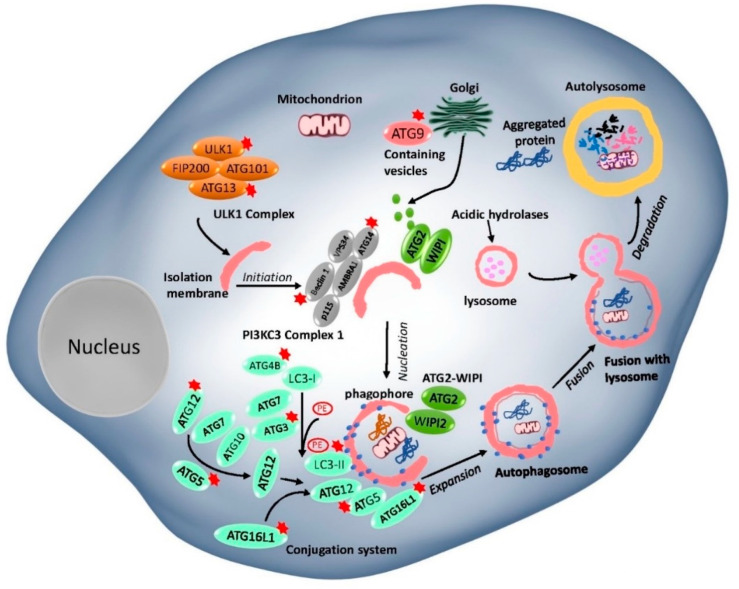
The mammalian core machinery of autophagy and target genes for Hepatocellular carcinoma (HCC) treatment. The core machinery comprises of ATG proteins formed five functional groups. (I) The ULK1 complex, consisting of ULK1, RB1-inducible coiled-coil protein 1 (FIP200), ATG101, and ATG13, which negatively regulates the mTOR complex. (II) The Beclin 1-class III PI3K complex consisting of Beclin 1, VPS34, P115, AMBRA1, and ATG14, controls the nucleation step of autophagosome formation. (III) The two ubiquitin-like conjugation systems (the ATG12-ATG5 system and the LC3 system). (IV) The WIPI1/2 and ATG2 complex. (V) The ATG9 retrieval complex. The targeted ATG genes in the autophagy core machinery in this review have been indicated by an asterisk (*).

In general, the basic autophagy process consists of the following steps: induction, vesicle nucleation, autophagosome, and autolysosome formation and degradation [69,70]. ATG proteins, which play an important role in autophagosome formation and the lysosomal delivery of autophagic cargo, are divided into five complexes (Figure 1): (I) Unc-51-like kinase 1 (ULK1) complex- ULK1, RB1-inducible coiled-coil protein 1 (FIP200), ATG101, and ATG13; (II) class III PI3K (PI3KC3) complex, the catalytic subunit vacuolar protein sorting 34 (VPS34), Beclin 1, and p115, joined by ATG14 or UV radiation resistance-associated gene protein (UVRAG); (III) two ubiquitin (Ub)-like proteins and conjugation systems: the ATG12-ATG5–ATG16L conjugation complex and Ub-like ATG8 family proteins (ATG8s), which form conjugates with membrane-resident phosphatidylethanolamine (PE); (IV) ATG18/WIPI (WD repeat domain phosphoinositide-interacting) proteins and ATG2; and (V) ATG9, a sole multi-spanning transmembrane protein which is involved in vesicle trafficking [70,71,72].

Among these complexes mentioned above, the mechanistic target of rapamycin complexes (mTORC1 and 2) is the most critical upstream autophagy regulator. mTORCs play an important role in regulating various cell biological functions, including but not limited to cellular growth, cell progression, cell migration, protein synthesis, and physiology and metabolism pathways [73]. Under nutrient-rich conditions, ATG13 and ULK1/2 are phosphorylated by mTOR, which inversely correlates with FIP200 phosphorylation to inhibit autophagy. Under nutrient deprivation, the mTORC1-dependent phosphorylation sites in ULK1/2 are rapidly dephosphorylated, allowing ULK1/2 to phosphorylate, and activate Atg13/FIP200, thereby inducing autophagy [74]. Activated Atg1/ULK1 complex further regulates the activity of the class-III phosphatidylinositol 3-kinase (PI3K) complex in sequence. Following this, two ubiquitination-like conjugation systems—namely, ATG12-ATG5-ATG16L and ATG8 systems—are involved in an autophagosome membrane elongation step. ATG7 (E1-like enzyme) and ATG10 (E2-like enzyme) proteins promote ATG12 and ATG5 conjugation, followed by the addition of ATG16L protein to the complex [75]. The second conjugation ATG8 proteins are covalently conjugated to lipid phosphatidylethanolamine (PE). LC3-PE proteins are cleaved by ATG4 at their C-terminus to generate a free cytosolic form of the protein LC3-I. LC3-I conjugated to a PE, resulting in the LC3-II form which is commonly used as a marker of autophagy [76]. ATG18/WIPI complex plays an important role in autophagosome formation by recognizing PI3K to phosphatidylinositol triphosphate (PI3P) at the nascent autophagosome [9]. ATG9A is a pass transmembrane protein that has been shown to have an essential role in autophagosome formation. It is found that ATG9A supplies inner components, such as proteins and lipids, to the autophagosome membranes via the trans-Golgi network [9]. After completing the whole process with the help of the protein complex, the cargo inside the autophagosome is delivered to the autolysosome and degraded by hydrolytic enzymes’ action. Accordingly, a better understanding of the underlying mechanisms in autophagy is essential before deciding to either induce or inhibit autophagy from treating human diseases in the future.

### 2.2. The Role of Autophagy Core Machinery Genes in Liver Cancer as Diagnostic Marker

Currently, HCC patients exhibit a high recurrence rate and poor survival [16,77]. It is important to discover convenient biomarkers to predict treatment risk and prognosis for HCC patients. Some core Atgs can be used as predictive biomarkers for the prognosis of HCC, and using autophagy-related markers will benefit HCC prognosis and extend therapeutic approaches [15].

Many reports have revealed the controversial role of LC3 expression in the clinicopathological features in HCC patients. For example, HCC tissues express higher levels of LC3A compared to adjacent non-tumorous tissue. A survival rate analysis showed a significant association between LC3A expression and poor prognosis [78]. However, another report has demonstrated that the lower expression or absence of LC3 is strongly linked to immediate mortality for HCC [79]. Further, a meta-analysis showed that positive LC3 expression was positively correlated with tumor size and overall survival. Therefore, the LC3 expression level may be related to the occurrence, evolution, and poor prognosis of liver cancer, suggesting that the LC3 could be a diagnostic marker associated with the occurrence and development of HCC [80].

Beclin-1 was taken as an independently predicted marker of HCC tumor progression. The Beclin-1 expression level was significantly associated to disease-free survival (DFS; *p* < 0.0001) and overall survival (OS; *p* < 0.0001) [81]. The study on 103 primary HCC patients showed that Beclin 1 expression was significantly lower in HCC tissues than adjacent tissues (72.8 vs. 89.5%, *p* = 0.015) [82]. A more recent study has confirmed reduced Beclin-1 and high HIF-1α expression associated with the development and progression of HCC [83]. Moreover, the expression level of ULK1 was significantly associated with tumor size and survival time [84].

The expression of LC3 and Beclin-1 were analyzed by immunohistochemistry on tissues from 190 HCC patients, and LC3 expression was a significant independent prognostic factor of overall survival (OS) and predict time to recurrence (TTR). Moreover, the expression of LC3 in the advanced stages of tumor-node-metastasis TNM (III) was correlated with a more prolonged survival [85]. Similarly, LC3B combined with ULK1 improved prognosis assessment in HCC patients [86]. Accordingly, LC3 can be used as a promising prognostic marker in HCC alone and/or combined with other related autophagy related genes may improve clinical diagnostic benefits.

P62 was not found in non-tumor areas and cirrhotic nodules but was found in 20 out of 20 (100%) tumor specimens. Induction of autophagy by Torin 1, a mTOR inhibitor, abolished the expression of p62, while inhibition of autophagy increased the expression of p62 in HCC cells in vitro. These results demonstrated that p62 expression change caused by autophagy level manipulation and further indicates that p62 may serve as a novel diagnostic marker for HCC [87].

An extensive bioinformatic analysis showed that 63 gene-related autophagy processes have different expression patterns between normal and HCC tissues. Based on the LASSO Cox regression algorithm, seven autophagy related genes (ATG9A, ATG7, RAB7A, GNAI3, CAPN10, EIF2S1 and SPNS1) were identified to be closely associated with the OS of HCC. They could be potential markers for prognostic risk signatures [88].

However, the methods for monitoring autophagic activity are complicated and elevated autophagic protein levels do not always directly correlate with increased autophagic activity. In terms of the diagnostic marker selection in HCC treatment, the autophagic protein level changes combine with the autophagic structures detection as well as the autophagic flux monitor may provide a more comprehensive and robust strategy.

## 3. The Role of Autophagy in Liver Cancer via Modulating Signaling Cascade

Two important mechanisms are involved in HCC molecular pathogenesis: (1) multiple molecular factors related to the cirrhosis induced by hepatitis infection, environmental or metabolic influences [89]; (2) mutations occurring in oncogenes or tumor suppressor genes [90,91]. These two mechanisms are closely related to several critical cell signaling pathways that influence HCC initiation and development. In our previous review, we mainly discussed the dual role of autophagy in HCC by elaborating the tumor-suppressive activity of ATGs, the role of p62 in liver tumorigenesis, and cancer cell survival and cell death mechanisms. In the following section, we will focus on the signaling cascades namely, PI3K-AKT-mTOR, AMPK-mTOR, the epidermal growth factor (EGFR) and insulin-like growth factor (IGF), mitogen-activated protein kinases (MAPKs), Wnt/β-catenin, p53, NF-κB and Nrf2-p62 pathways. We will also discuss the effect of autophagy on HCC via crosstalk with the pathways above and potential therapeutic application in HCC therapy.

### 3.1. The PI3K-AKT-mTOR Pathway

In cancer and particularly in HCC, the PI3K-Akt-mTOR pathway plays an important role in regulating cell growth, proliferation, apoptosis and angiogenesis [92,93,94]. The PI3K-Akt-mTOR pathway is highly activated in 15–41% of HCCs, and inhibition of mTOR plays an antitumor role in HCC [95].

IGF-1 activates PI3K and, in turn, activates AKT, which proceeds to phosphorylate. Inactivated Ras Homologue Enriched In Brain (RHEB) and Tuberous Sclerosis Complex 2 (TSC2) will stimulate mTORC1 activation [96], leading to the inhibition of autophagy [97]. In contrast, with stress conditions such as low nutrition and hypoxia, this pathway is inactivated and cell growth and proliferation are suppressed via induction of autophagy [97,98].

Over-expression of SOCS5, a member of the suppressor of cytokine signaling (SOCS) protein family, can enhance the cell invasion and migration via suppression of PI3K-Akt-mTOR-mediated autophagy in HCC in vitro. On the other hand, inhibition of SOCS5 suppressed cell migration and invasion by activating PI3K-Akt-mTOR-mediated autophagy in HCC. Combined inhibition of mTOR and SOCS5 may provide a potential therapeutic approach for metastasis inhibition in HCC patients [99].

As a tumor suppressor gene, PTEN mutation is involved in the process of initiation and progression in HCC [63,100]. PTEN deletion is frequently detected in 5% of HCCs [101], while over-expression results in the tumor size reduction and further activation of the PI3K-Akt-mTOR pathway [102]. Long noncoding RNA (lncRNA) HULC promotes liver cancer development via increasing cellular autophagy, which activates the AKT-PI3K-mTOR pathway via inhibiting PTEN in HCC cells [103]. This observation revealed autophagy plays a pro-oncogenic role by utilizing the PTEN-mTOR pathway.

### 3.2. The AMPK-mTOR Pathway

The adenosine monophosphate (AMP)-activated protein kinase (AMPK)-mTOR pathway plays an important role in cell growth, cell proliferation and metabolism as well as autophagy regulation [104]. More and more studies showed that the AMPK-mTOR pathway is involved in liver cancer metabolism and tumorigenesis [105,106]. Under stress conditions, ATP reduction would enlarge the AMP/ATP ratio and further activate the energy-sensing kinase, liver kinase B1(LKB1), and AMPK. In addition, TSC2 and Raptor (the regulatory associated protein of mTOR) can be phosphorylated by AMPK, leading to the inactivation of mTORC1 and the induction of autophagy [107,108]. Under glucose starvation, AMPK can promote autophagy by direct phosphorylation of ULK1 [109,110].

Recent studies have highlighted the tumor suppression role of AMPK in HCC. The activation of AMPK was found to be associated with the inhibition of cell motility, invasiveness and subsequent cell apoptosis in HCC cells [111,112]. Interestingly, AMPK also induces autophagic and apoptotic cell death by activating transcription factor CCAAT/enhancer-binding protein delta (CEBPD) and enhancing LC3B expression in HCC [113]. Contrarily, loss of AMPK in HCC cells may promote cell progression, cell survival, migration, and invasion via different oncogenic molecules and pathways suggested that activation of AMPK may possess potential anti-HCC function [114].

### 3.3. The EGFR and IGF Pathway

EGF receptor (EGFR/HER1) is one of the most relevant growth factor receptors in HCC. It plays a vital role in tumor proliferation and angiogenesis by activating the PI3K-AKT-mTOR and RAF-MEK-ERK pathways [115]. A high expression of EGFR is detected in adult hepatocytes suggesting that EGFR plays an essential role in hepatoprotective and pro-regenerative functions of the liver [116]. Moreover, the EGFR signaling pathway is also involved in proliferation, survival, and tumorigenicity in vitro in human HCC cell lines [117]. The genetic over-expression of TGF-α or EGF enhanced the incidence of HCCs in mice [118]. Importantly, the inhibition of EGFR with the tyrosine kinase inhibitor, gefitinib, exhibited an antitumoral effect in diethylnitrosamine (DEN) induced HCC rats suggesting that gefitinib is a potential drug for the HCC treatment [119].

The IGF signaling pathway plays a pivotal role in antiapoptotic, the stimulation of proliferation, the activation of angiogenesis, and the initiation and maintenance of oncogenesis [120]. The crucial role of this pathway, as well as each of its components, have been demonstrated in the carcinogenic and metastatic potential of HCC both in vitro and in vivo [121]. In HCC, dysregulation of IGF signaling mainly occurs at the level of IGF2 bioavailability. The over-expression of IGF2 can be detected in 16–40% of human HCCs and several HCC animal models [122,123]. The IGF2 gene is transcribed in a developmental- and tissue-specific way and four promoters (Pl–P4) were involved in IGF2 expression level change [124]. The disruption of the IGF2 promoter regulation is a common feature of human HCCs suggested that the frequent loss of biallelic IGF2 expression as a result of the loss of P1 activity may potentially be used as a diagnostic or monitoring marker for human HCC [125,126]. Unlike the high-level expression of IGF-2 in human HCCs, a lower level of IGF2R is found in approximately 80% of HCCs [127]. This excess ligand can enhance the receptor binding capability and MAPK and PI3K-AKT-mTOR pathways which are master regulators in autophagy modulation [101].

### 3.4. The MAPK Pathway (ERK, JNK, p38)

Mitogen-activated protein kinases (MAPKs) pathways are the major signaling systems involved in cell fate decisions such as proliferation, differentiation, and cell death. Mammalian MAP kinases are divided mainly into three groups based on their structure and function: (1) c-Jun N-terminal kinase or stress-activated protein kinase (JNKs or SAPKs), (2) extracellular signal-regulated kinases (ERKs), and (3) the p38 MAPK [128]. The MAPK axis is one of the best-characterized pathways in HCC and Ras, Raf, MEK and upstream kinases of the ERK MAPK pathway, which is activated by several ligands such as HGF, IGF, VEGF, and PDGF [129].

The RAF-MEK-ERK cascade plays a critical role in cell proliferation, differentiation, angiogenesis and survival [130]. Currently, the activation of the RAF-MEK-ERK pathway in hepatocarcinogenesis can be summarized as follows [131]. Firstly, oncogenic mutations within the *RAS* gene results in constitutive pathway activation through c-RAF [130]. *C-RAF* is overexpressed in all 30 HCC tissue samples, suggesting that *CRAF* activation may play a critical role in HCC [132]. In the Catalog of Somatic Mutations in Cancer (COSMIC) database (update to June 2020), *B-RAF* exhibits a high mutation rate of (5.8% in tested samples), while mutations within the gene *NRAS* (1.1%) are rare in HCC [133]. The COSMIC database shows that the NRAS gene mutation is less than the KRAS mutation (3.5%) [134]. Secondly, modulation of expression of growth factors and their receptors contributes to the CRAF activation and downstream RAF-MEK-ERK activation [130]. In addition, down-regulation of the MAPK negative regulatory proteins phosphatidylethanolamine binding protein 1 (RKIP) and the Ras inhibitors Sprouty (Spry)/Spred proteins is often observed in human hepatocarcinogenesis [135]. Finally, hepatitis B and C viruses can utilize the RAF-MEK-ERK pathway to regulate hepatocyte survival and viral replication via autophagy induction [136,137]. For HBV infection, HBx activates Raf-MEK-ERK signaling cascade during hepatocarcinogenesis [138], while for HCV, the core protein activates the Raf-MEK-ERK signaling cascade in HCC [139]. 

### 3.5. The Wnt/β-catenin Pathway

The canonical Wnt pathway regulation is caused by the binding between Wnt proteins and cell-surface receptors of the Frizzled family [101], resulting in the activation of downstream effector Disheveled, which consequently prevents the phosphorylation of β-catenin, promotes the translocation from the cytoplasm to the nucleus [140]. The Wnt/β-catenin pathway is one of the most common disrupted pathways in HCC and activation of the Wnt pathway had been found in approximately 30% of HCC [141]. A study demonstrated that the level of autophagy was inversely correlated with Dishevelled Segment Polarity Protein 2 (Dvl2) expression and activation of Wnt signaling in tumor cells [142].

The β-catenin mutation is detected in 12–26% of HCCs, and these mutations are closely related to chronic HCV infection, which is important for HCC initiation [143]. In addition, the accumulation of β-catenin in the cytoplasm and nucleus is found in 50–70% of HCC although the accumulation alone does not transform the tumor from benign to malignant [144]. Interestingly, several Wnt/β-catenin signaling genes play a role as tumor suppressors in HCC development. For example, *Iqgap2* deletion promotes β-catenin activation and the tumorigenesis of HCC [145]. *Sox17* can inhibit human HCC cell growth by negatively regulating the canonical Wnt/β-catenin signaling pathway [146], while as a tumor suppressor, *GABARAPL1* inhibits Wnt signaling by the mediation of Dvl2 degradation suggested that the inhibition role of *GABARAPL1* on the Wnt signaling is autophagy dependent [147].

Interestingly, the suppression of cell growth has been found when cells were treated with the IWP12 porcupine inhibitor or knockdown of Wntless (WLS) to block Wnt secretion in HCC. However, the Wnt secretion level change does not affect the β-catenin signaling in most HCC cells, suggesting that other possible mechanisms involved are essential in cell growth reduction. These results indicated that mutations of critical components in the Wnt pathway and the β-catenin level do not strongly rely on extracellular Wnt ligand in most liver cancers. Hence, the suppression of Wnt secretion reduces cell growth of HCC independent of β-catenin signaling [148].

### 3.6. The p53 Pathway

p53 mutation had been found in more than 50% of aflatoxin B1-induced HCC, around 45% of HBV-related HCC and about 13% of HCV-related HCC [149]. Currently, different types of p53 were used as the target for therapeutic strategies in HCC [150]. Many discoveries had shown that autophagy suppresses p53, and p53 activates autophagy [151].

On the one hand, when cancer cells express fully functional wildtype p53, they can recover from physiological apoptosis while suppression of p53 may promote tumor. Injection of recombinant adenovirus p53 (rAd-p53) with transarterial chemoembolization (TACE) treatment is effective and safe for treating of unresectable HCC. This p53 gene therapy-based TACE improves the overall survival (OS) and PFS rates compared to TACE monotherapy [152]. Walsuronoid B, a limonoid compound extracted from Walsura robusta, inhibited cell proliferation and induced apoptosis via the ROS-p53 signaling pathway in HepG2 and Bel-7402, showing that Walsuronoid B possesses potential anti-cancer function by the upregulation of p53 levels [153].

On the other hand, some mutants of p53 play new oncogenic functions referred to as “gain-of-function” which are different from their original function and effect of wildtype p53. Hence, strategies to inhibit these new functions than restore wildtype p53 have proven effective. PRIMA-1 can restore and stabilize the original DNA binding domain of p53 and refold mutant p53 in HCC cell lines [154]. RETRA elevates TAp73 expression by disruption of the TAp73/mutant p53 complex and promotes the releasing ability of TAp73. Next, the released p73 is involved in many cell-cycle arrest and apoptosis processes by activating the target genes of p53 [155].

### 3.7. The Nuclear Factor-κB (NF-κB) Pathway

Interestingly, the inhibition of NF-κB may contribute to both beneficial and negative effects on hepatocyte viability. NF-κB acts as a two-edged sword in hepatocarcinogenesis, where suppression of NF-κB increasees liver injury. Hence, the effects of NF-κB on HCC strongly depend on the cell type and the status of NF-κB activation. A high level of NF-κB expression in non-parenchymal cells generally promotes inflammation and HCC. However, the activation of NF-κB in parenchymal cells shows both suppression and promotion role in HCC [156].

NF-κB shows different specific effects on different cell types such as hepatocytes, Kupffer cells, and HSCs and/or hepatic myofibroblasts [156]. The IL-1α released from dying hepatocytes can increase hepatic injury and stimulate regenerative responses in progenitor cells and activation of Kupffer cells. Activated HSCs and/or hepatic myofibroblasts produce an extracellular matrix; increased extracellular matrix is associated with HCC progression [157]. NF-κB activation occurs early stage of HCC with viral or nonviral etiologies and has been associated with the acquisition of a transformed phenotype during hepatocarcinogenesis [158]. Many factors may cause NF-κB activation. For example, oncogenic HBV-X protein activates the NF-κB pathway via the upregulation of TANK-binding kinase 1(TBK1) in HBV-induced HCC [159]. Moreover, patients with advanced liver disease with high levels of LPS or fatty acids resulting in activation of NF-κB [160].

NF-κB (nuclear factor kappa-light-chain-enhancer of activated B cells) as a protein complex, is a key transcriptional regulator of inflammation and plays an important role in modulating the immune and infection response in the liver [161]. In human liver cancer, activated NF-κB is found very frequently and occurs at the early stages of hepatocarcinogenesis, mainly caused by viral or nonviral factors [158,162].

A high level of mitochondrial fission is detected in HCC tissues and significantly contributes to the poor prognosis of liver cancer patients. In addition, increased mitochondrial fission promotes autophagy and cell survival via elevated ROS production and AKT activation, which facilitates transcriptional activity of NFκB in liver cancer cells, while the inhibition of mitochondrial division significantly suppressed tumor growth in vivo [163]. The over-expression of Dynamin-1-like protein (Drp1) increased mitochondrial fission and promoted cell proliferation by facilitating G1/S phase transition and autophagy induction in vitro and in vivo. Moreover, the Drp1-mediates mitochondrial fission was affected by p53/p21 and NF-κB-cyclins, indicating modulation of mitochondrial fission by targeting Drp1 may give us a clue for searching the novel therapeutic strategy for HCC treatment [164].

### 3.8. The Nrf2-p62 Pathway

Phosphorylation of p62/Sqstm1 at Ser349 enhanced the tolerance of cells to anti-cancer drugs and proliferation potency via the activation of the transcription factor Nrf2. Further study showed that inhibition of phosphorylated p62-dependent Nrf2 activation by *N*-[2-acetonyl-4-(4-ethoxybenzenesulfonylamino) naphthalene-1-yl]-4-ethoxybenzenesulfonamide (named as K67) suppresses the proliferation and drug resistance of HCC. Therefore, K67 might take as a drug against HCC cells resistant to chemotherapy in a p62 phosphorylation manner [165,166]. p62 is an important autophagy marker to indicate the protein accumulation and degradation. By interaction with Keap1/Nrf2, which play an important role in oxidative stress regulation, p62 is involved in cell survival, growth and cell death pathways in HCC. Currently, targeting p62 or the Keap1/Nrf2 system with related pathways, such as autophagy, is a potential therapeutic strategy in HCC therapy [167]. More detailed molecular mechanisms underlying the HCC therapeutic perspective in terms of targeting Nrf2-p62 via autophagy have been discussed and reviewed recently [168].

## 4. Targeting the Autophagy Core Machinery for Liver Cancer Treatment

Sorafenib is a kinase inhibitor drug approved for the treatment of primary HCC. Treatment of HCC cells with sorafenib increased LC3-II and decreased the expression of p62 which suggests that autophagic flux is increased. However, inhibition of autophagy by autophagy inhibitor (Baf-A1) and ULK1-silencing resulted in increased cell death and enhanced the sensitivity of HCC cells to sorafenib. Furthermore, silencing *Ulk1* suppressed the tumor growth in a xenograft mouse model. The inhibition of autophagy by XST-14, a ULK1 inhibitor, can significantly reduce the HCC progression. These data suggest that targeting ULK1 could be used as a novel therapeutic strategy in HCC treatment, especially in combination with sorafenib [169].

Furthermore, besides the application in biomarkers, modulation of members of core molecular machinery Atgs may exert direct effects on HCC therapy. The expression of miR-26a/b inhibited autophagy by targeting ULK1 and promoted cell death in HepG2 cells while overexpression of miR-26a/b sensitized hepatomas to Dox treatment via the inhibition of autophagy in vitro and xenograft nude mice models. [65]. In addition, miR-375 negatively regulated sorafenib-induced autophagy by direct targeting ATG14 and enhanced the sensitivity of HCC to sorafenib [170].

The over-expression of lncRNA HANR in sorafenib-resistant HCC tissues and cells inhibit sorafenib susceptibility of HCC cells by promoting autophagy. miR-29b reduces HANR-induced sorafenib resistance by targeting HANR and suppressing autophagy in HCC cells. Meanwhile, HANR could act as a competing endogenous RNA (ceRNA) to upregulate ATG9A expression by inhibiting miR-29b expression. miR-29b enhances sorafenib sensitivity by inhibiting autophagy, which is caused by direct targeting ATG9A in sorafenib-resistant HCC cells, indicating that the suppression of autophagy via miR-29b/ATG9A axis may be a potential target to prevent sorafenib-resistant in HCC [171]. Similarly, a study in lncRNA nuclear enriched abundant transcript 1 NEAT1 showed that NEAT1 lower sorafenib efficacy and induced autophagy in liver cancer cells. As a ceRNA, NEAT1 could upregulate ATG3 expression by sponging miR-204 levels. Accordingly, this result suggested that suppression of autophagy via modulating NEAT1/miR-204/ATG3 signaling may be a good option for sorafenib-induced chemoresistance in HCC treatment [172].

Under hypoxic conditions, the downregulation of autophagy using 3MA and silencing of Atg4B, which involved PI3KC3 inhibition and the suppression of the conversion of LC3-I to LC3-II, inhibited the proliferation of HCC cells. Moreover, the intracellular ATP level in autophagy deficiency HCC cells was lower than in non-treated cells due to the impairment of mitochondrial b-oxidation. This study revealed the protective role of autophagy that involves the proliferation of HCC cells by activating mitochondrial b-oxidation [173].

Increased apoptosis, inflammation, and fibrosis were detected in hepatocyte-specific *ATG5* knockout mice, caused by aberrant polyubiquitinated proteins accumulation. However, these pathological changes were markedly abolished in *Nrf2* and *ATG5* double knockout mice. These results suggest that loss of autophagy causes cell death, which will further result in liver inflammation and tumorigenesis [174].

The higher level of lncRNA HAGLROS was found in HCC tissues and correlated with the progression and development of HCC. Suppression of HAGLROS upregulated the miR-5095 expression, which further suppresses apoptosis and autophagy by targeting ATG12 [175]. The high level of miR-135a is associated with decreased disease-free survival in HCC. The autophagic processes were inhibited by ectopic expression of miR-135a via targeting Atg14. These results suggested that miR-135a plays a novel function in the regulation of autophagy, which could be used as a potential target for HCC treatment [176].

The inhibition of autophagy (with 3MA or ATG5/Beclin1 knockdown) results in the enhancement of apoptotic cell death induced by a number of agents, including physicon [177], fangchinoline [178], Bcl-2 inhibitor ABT-737 (which induces autophagy via JNK activation and the dissociation of Beclin 1 from the Beclin 1/Bcl-2 complex) [179], AKT inhibitor 1/2 (AKTi-1/2) in vitro and in vivo [180], doxorubicin [181], indicating that autophagy inhibition could be an effectual approach in combination therapy to overcome chemoresistance in HCC. Moreover, the study also revealed that the BMP4 induced autophagy was mediated through activating the JNK1/Bcl2 pathway [182]. Similarly, inhibiting autophagy by using autophagy-specific inhibitors (3-MA, chloroquine, and bafilomycin A1) or *Beclin1* and *ATG5* silencing, enhances apoptosis induced by naturally occurring products such as Arenobufagin, a bufadienolide from toad venom [183,184].

The silencing of LC3 with siRNA results in the down-regulation of LC3 and autophagosomes, resulting in increased apoptosis and angiogenesis potential in SMMC7721 HCC cells and HCC xenograft model [185], while suppressing LC3 by lentivirus-mediated shLC3 silencing inhibits serum deprivation-induced autophagy and significantly reducing cell proliferation and blocking cell cycle progression by increasing of the percentage of G1 (2N DNA content) phase cells in HepG2 cells. Combined treatment with shLC3 and epirubicin, an anthracycline drug used for chemotherapy, significantly decreased the survival rate of HepG2 cells, compared with epirubicin alone suggesting that LC3 plays a key role in HCC tumorigenesis, and is a potential therapeutic target for HCC [186].

## 5. Targeting Autophagy for Liver Cancer Treatment via Signaling Cascades

As discussed above, the PI3K/AKT/mTOR pathway plays an important regulatory role in liver tumorigenesis and HCC therapy via autophagy modulation. Due to the double-sided role of autophagy in HCC, the significance of balanced autophagy activity in PI3K/AKT/mTOR pathway in HCC treatment has become incredibly important [187]. Studies have shown that the abnormal activation of AKT and PI3K in cancer cells, leading to the activation of PI3K/AKT/mTOR pathway, which will regulate cancer cell proliferation by the activation of protein synthesis via p70S6K and 4EBP1 phosphorylation [188]. Hence, the inhibition of the PI3K/AKT/mTOR pathway may benefit cancer therapy. However, the inhibition of PI3K/AKT/mTOR pathway could also activate autophagy, promoting cell perforation in the later stage of cancer. This evidence suggested that inhibition of the PI3K/AKT/mTOR pathway may lead to a conflicted role in HCC treatment. Therefore, the combination of PI3K/AKT/mTOR pathway inhibition and autophagy inhibitors can inhibit hepatoma proliferation [64]. On the other hand, the activation of autophagy does not promote tumor progression in the late stage of HCC. Over-activation of autophagy by inhibition of PI3K/AKT/mTOR pathway may cause autophagic cell death in liver cancer [189]. Accordingly, to investigate the controversial function of PI3K/AKT/mTOR in HCC the different stages of HCC development may be considered carefully.

The induction of autophagy by the over-activation of the AMPK/mTOR pathway promotes autophagic cell death and inhibits hepatoma cell growth [190,191]. Autophagy activation can also help cells survive under stress conditions such as starvation, hypoxia, and chemotherapy [192]. Hence, reports also reveal that the inhibition of autophagy may promote cell death by enhancing the sensitivity of cancer cells to cytotoxic drugs [15]. In summary, the double-sided effects of the role of AMPK/mTOR in liver cancer also need to be highlighted.

Thus far, studies on the role of the Ras/Raf/MEK/ERK pathway in liver cancer via autophagy modulation are limited. Recently, evidence has indicated that Ras/Raf/MEK/ERK pathway plays an important role in liver cancer via regulation of autophagy by mediating mTOR [193]. Of note, mTOR acts as the major regulator involved in PI3K/AKT, AMPK, Ras/Raf/MEK/ERK pathways. These three mTOR-mediated pathways show multiple crossing points and are not independent parallel pathways.

The abnormal activation of Wnt/β-Catenin signaling has been found in 20% to 90% of liver cancers [194]. In terms of modulation of Wnt/β-Catenin in liver cancer treatment, there two points need to be highlighted. Firstly, some oncogenes, such as H-Ras proteins, have been found mutated simultaneously with β-Catenin and increases the incidence of liver cancer to 100% in mice [195]. Thus, the inhibition of Wnt/β-Catenin signaling may also be combined with other oncogenic inhibitors to improve the anti-carcinogenesis outcome in liver cancer. Secondly, combined therapy targeting the Ras/Raf/MAPK and Wnt/β-catenin pathway can suppress cell proliferation in HCC [196,197]. In this context, combined therapy may be a promising strategy to overcome the complex network of signaling pathways. It is important to identify the selective inhibitors of this pathway and eventually benefit the patient with advanced HCC.

p53 mutation is most commonly presented in human tumors, including liver cancer. Autophagy can be activated or inhibited by p53 indicating the dual function of p53 in the modulation of autophagy in HCC. On the one hand, wildtype nuclear p53 can be taken as the transcription factor which induces pro-autophagic genes under stressed conditions. On the other hand, cytoplasmic p53 represses autophagy. Notably, in contrast to nuclear p53, cytoplasmic p53 inhibits AMPK, a positive regulator of autophagy, and activates mTOR to suppress autophagy. Besides, in the absence of p53, autophagy can be induced, and wildtype p53 promotes cell death in HDACi such as SAHA-treated HCC [198]. The mutational status of the tumor cell and the mechanisms of p53 involved in modulating autophagy and apoptosis need further investigation.

The role of NF-κB in inflammation and cancer provides a high clinical relevance for HCC. However, the gap between the basic research results and the application of the chemical compound remains investigated. The principle for the design of pharmacologic therapies targeting NF-κB should follow the direct conclusion from different model systems and circumstances [156]. NF-κB inhibition with increased liver injury promotes liver cancer development in the IKKγ knockout mice, TGF-β-activated kinase 1 (TAK1) knockout mice, and DEN-treated IKK2 knockout mice. However, under less liver injury conditions, inhibition of NF-κB can promote cell death when the producing tumor-promoting proinflammatory mediators are targeted. Increasing evidence showed that NF-κB involved in the initiation and promotion of liver cancer development in the chronically inflamed liver. These studies suggested that compounds targeting of NF-κB pathway may consider the stages of liver cancer and the inflammatory status.

As an autophagy substrate, p62 is degraded during autophagy activation. Meanwhile, p62 can also inhibit autophagy by activating mTORC1 [199]. Moreover, it is well known that mTORC1 induction promotes liver cancer development. Therefore, p62-mediated mTORC1 activation may also contribute to liver tumorigenesis. Accordingly, these findings suggested that p62 may function as a tumor promoter role by modulating multiple signaling pathways including mTOR and autophagy.

In general, either the induction or suppression of autophagy could benefit the HCC treatment by targeting signaling pathways. Currently, many chemicals and drugs have been used in vitro or in vivo and clinical trials in HCC therapy. We hereby summarized these compounds to clarify the dual role of autophagy inducers and inhibitors in liver cancer (Table 1).

## 6. Conclusions and Future Directions

**Figure 2:**
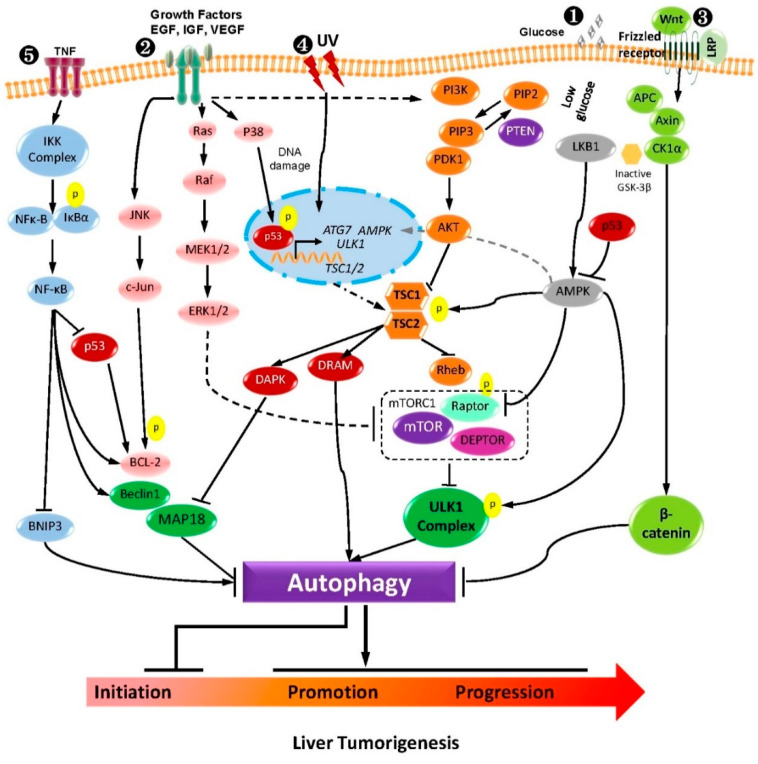
The crosstalk between the major signaling pathway regulating autophagy and liver tumorigenesis. (1) PI3K-AKT-mTOR and AMPK-mTOR pathway receptor tyrosine kinases promote the conversion of PIP2 to PIP3 and the activation of PI3K. Activation of AKT by dephosphorylation of PIP3 caused by PTEN could negatively regulate PI3K signaling. Amino acids and nutrient-rich conditions initiate mTORC1 activation. In comparison, starvation and oxidative stress inhibit mTORC1 activation and induce autophagy. AMPK regulates autophagy either by phosphorylating TSC via the mTOR pathway or by directly phosphorylating ULK1. (2) EGFR, IGF, and MAPK pathway EGFR family members contribute to autophagy by activating three of the major signaling pathways for cell survival via the regulation of the autophagy process JNK/c-Jun, Ras/Raf, and the PI3K/AKT pathway. (3) Wnt-β-catenin pathway: This pathway results in the activation and nuclear recruitment of β-catenin protein, leading to directly regulating autophagy. In the canonical Wnt pathway, without Wnt β-catenin is degraded by a destruction complex and would not accumulate in the cytoplasm. This destruction complex consists of the following proteins: Axin, adenomatosis polyposis coli (APC), protein phosphatase 2A (PP2A), glycogen synthase kinase 3 (GSK3), and casein kinase 1α (CK1α). (4) p53 pathway: Autophagy is also mediated by nuclear p53 activity, a transcription factor in stress conditions, such as UV, etc. p53 primarily induces the canonical pathway of autophagy by PI3K-AKT-mTOR, AMPK-mTOR, and ULK1 complex. Alternatively, damage-regulated autophagy modulator (DRAM), death-associated protein kinase (DAPK), or ATG7 upregulation by the p53 can also initiate autophagy. (5) NFκ-B pathway IKKα/β and NF-κB induce autophagy by increasing the Beclin 1 and other autophagy-related proteins’ expression levels. Moreover, increased autophagy can degrade IKK components and repress autophagy. Besides this, NF-κB signaling could inhibit autophagy by the over-expression of autophagy repressors, such as Bcl-2/Xl and BNIP3.

As outlined in this review, HCC is a notoriously difficult disease to treat by traditional approaches. Until now, only a few drugs have been approved for clinical treatment. Research on HCC development and treatment has focused on the molecular and cellular signaling mechanisms. Since autophagy is a highly selective and powerful process that is critically implicated in various fundamental cellular processes, it plays a critical role in hepatocarcinogenesis and may be regulated through several key signaling pathways (Figure 2). Since considerable pathways contribute to HCC development, targeting single molecules or pathways may have a limited benefit in HCC therapy. The use of a traditional combination regimen can lead to tolerability and drug resistance. Therefore, an unbiased re-evaluation of therapeutic strategies is required for HCC treatment. The key points for future treatment in HCC will be highly dependent on the underlying molecular pathways involved in autophagy. Our primary task aims to map the context-dependent functional networks in which autophagy and HCC therapy are embedded. The fact that the crosstalk of autophagy with many signaling pathways may give some idea of the landscape of this task.

In this review, we need to think about the following questions to be solved in the future: Firstly, how to select the diagnostic markers properly before or during the HCC treatment? Given the complications of the HCC tumorigenesis process, we may need to decide where and when to look at the markers. Moreover, a combination of several markers may provide a solid result compared with using a single marker. Secondly, in terms of the combination therapy using different autophagy inhibitors such as CQ/HCQ with sorafenib or other small molecules, we need to elucidate the underlying molecular mechanisms and cytotoxin of those chemicals and the specific role to autophagy before we proceed on to the next step. Thirdly, these pathways are the most important signaling cascades that are tangled with liver cancer development and presented complicated molecular mechanisms. Additionally, autophagy can affect liver development on multiple levels in most pathways. These findings further enhance the notion that autophagy shows the multi- nature of tumorigenesis. Hence, when and how to block or activate the related pathways will open up the possibilities for liver cancer treatment. Finally, it is important to find the specific inducers and inhibitors of autophagy. How to balance the cell death and cell survival mechanisms caused by autophagy is of utmost concern in treating HCC. Another balance we need to consider is how to target the right stage of HCC development. Hence, facing the contrasting roles of autophagy in liver cancer, we need to select the proper way to achieve better therapeutic benefits, induction or inhibition of autophagy is all dependent on the particular case and analysis.

## Figures and Tables

**Table 1:** Targeting autophagy as an anti-cancer strategy for liver cancer treatment via signaling cascades.

Pathways Involved	Drugs/Compounds	Induction (+)/Inhibition (−) of Autophagy	In Vitro/In Vivo/Clinical Effects on HCC	References
PI3K-AKT-mTOR pathway	Baicalein	−	Synergized cell death in patient-derived xenograft (PDX) model.	[200]
	Long non-coding RNA LINC00160	+	Improved cell viability and tumorigenesis; silencing of LINC00160 suppressed HCC cell viability and tumorigenesis via suppression of autophagy.	[201]
	Rapamycin/everolimus (RAD001)	+	Displayed antiproliferative and anti-angiogenesis activities in HCC. Rapamycin induced a partial response, and the median OS was 6.5 in 24% of patients in a study with 21 advanced HCC patients.	[202,203,204]
	KRX-0401 (perifosine)	+	Tested in a phase II clinical trial which reported a median time to progression (TTP) of 3.2 months.	[205]
	mitoxantrone (MTX)	+	Inhibited cell growth and enhanced apoptosis in HepG2 cells.	[206]
	Lycorine	+	Induced autophagy in HCC cells and suppressed growth of xenograft tumors.	[207].
	NaHS	+	Significantly inhibited cell migration, proliferation and cell division, and induced cell apoptosis in HCC cells.	[208]
	Bicyclol	+	Effectively inhibited cell proliferation and inhibited cell growth in HepG2 cells.	[209].
	Barbaloin (Aloin) and Metformin (MET)	+	Inhibited cell proliferation, invasion, promoted apoptosis and suppressed the tumor growth in vitro and in vitro.	[210].
AMPK-mTOR pathway	RA-XII	−	Effectively inhibited HepG2 cell proliferation and enhanced cell death.	[211]
	Glycochenodeoxycholate (GCDC)	+	Promoted cell and tumor invasion via AMPK-mTOR.	[212]
	Cannabinoids (Δ^9^-THC)	+	Reduced growth of HCC xenografts, through inhibiting mTORC1 axis and AMPK stimulation.	[213]
	Britannin and Bigelovin	+	Significantly suppressed cell growth and HepG2 cancer xenograft growth.	[190,214]
EGFR and IGF pathway	Gefitinib and lapatinib	+	Completed phase II trials (NCT00107536, NCT00071994, NCT00101036) in advanced HCC. Modest antitumor activity for gefitinib at 250 mg daily; Lapatinib induced cytotoxicity and autophagic cell death.	[81,119,215]
	YM201636	+	Inhibited tumor growth without notable systemic toxicity in vivo.	[216]
MAPK pathway (ERK, JNK, P38)	Astragaloside II	−	Significantly inhibited autophagy and promoted 5-fluorouracil (5-FU)-induced cell death.	[217]
	Vitexin	−	Significantly suppressed cell viability via inducing apoptosis and inhibiting autophagy in SK-Hep1 and Hepa1-6 cells and inhibited tumor growth in vivo.	[218]
	Niraparib	+	Induced cytotoxicity and autophagy in response to its cytotoxicity in Huh7 and HepG2 cells.	[219]
	Xanthoangelol	+	Exhibited antitumor properties in HCC.	[220]
	β-Thujaplicin	+	Induced autophagic cell death and inhibited cell growth through ROS-mediated p38/ERK-MAPK signaling.	[221]
Wnt/β-catenin pathway	2,5-Dichloro-*N*-(2-methyl-4-nitrophenyl) benzenesulfonamide (FH535)	−	Effectively suppressed tumor progression by inhibition of the Wnt/β-catenin pathway and reduction in autophagic flux in a mouse xenograft model and HCC cells.	[222]
	Ginsenoside Rh2 (GRh2)	+	Suppressed cell growth via coordinated autophagy and β-catenin signaling in HCC.	[223]
	Ad.wnt-E1A(△24 bp)-TSLC1	+	Promoted autophagic cell death in HCC stem cells and inhibited growth of transplanted hepatic cancer stem cell (CSCs) tumors and extended survival period of mice.	[224].
p53 pathway	Dioscin	−	Inhibited proliferation and migration, apoptosis, autophagy by in SMMC7721 and HepG2 cells and inhibit ed primary tumorigenesis in xenografts.	[225]
	Tanshinone (TA) I	−	Induced cell apoptosis by suppressing p53/DRAM-activated autophagy in HepG2 and Huh7 cells.	[226]
	Matrine	+	Inhibited proliferation and induces apoptosis via induction of autophagy dependent on p53 inactivation in SMMC 7721 cells.	[227]
	Resveratrol	+	Inhibited proliferation and mobility of HCC cells.	[228]
Nuclear factor-κB (NF-κB) pathway	Ulinastatin (UTI)	−	Enhanced the outcome of liver cancer chemotherapy.	[229]
	Bisindolylmaleimides (BMA-155Cl	+	Induced apoptotic cell death in HCC cells.	[230]
